# Effects of advanced glycation end products on stem cell

**DOI:** 10.3389/fcell.2024.1532614

**Published:** 2024-12-24

**Authors:** Zetai Zheng, Hui Zhou, Wenwen Zhang, Tingyu Wang, Sathiskumar Swamiappan, Xinsheng Peng, Yanfang Zhou

**Affiliations:** ^1^ Department of Pathophysiology, Guangdong Medical University, Dongguan, China; ^2^ School of Pharmaceutical Sciences, Guangdong Medical University, Dongguan, China

**Keywords:** stem cell, cell functions, advanced glycation end products, rage, glycation

## Abstract

In recent years, stem cell therapy has become a pivotal component of regenerative medicine. Stem cells, characterized by their self-renewal capacity and multidirectional differentiation potential, can be isolated from a variety of biological tissues, including adipose tissue, bone marrow, the umbilical cord, and the placenta. The classic applications of stem cells include human pluripotent stem cells (hPSCs) and mesenchymal stem cells (MSCs). However, numerous factors can influence the normal physiological function of stem cells. For instance, in diabetes mellitus, advanced glycation end products (AGEs) accumulate in the extracellular matrix (ECM), impairing the physiological function of stem cells. These substances are closely associated with aging and the progression of numerous degenerative diseases. AGEs can create an environment that is detrimental to the normal physiological functions of stem cells. By binding to the primary cellular receptor for advanced glycation end products (RAGE), AGEs disrupt the physiological activities of stem cells. The binding of RAGE to various ligands triggers the activation of downstream signaling pathways, contributing to the pathophysiological development of diabetes, aging, neurodegenerative diseases, and cancer. Therefore, there is an urgent need for comprehensive research on the impact of AGEs on stem cells, which could provide new insights into the therapeutic application of stem cells in regenerative medicine.

## 1 Introduction

Stem cells (SCs) possess unique self-renewal capacity and multidirectional differentiation potential. They can be derived from various biological tissues, including bone marrow, adipose tissue, the umbilical cord, and the placenta. Additionally, SCs exhibit multiple functions, including nutritional support, migration ability, and immunosuppression, and hold broad potential for research and application in regenerative medicine (Naji et al., 2019). In regenerative medicine, treatment strategies focus on tissue repair and cell replacement. The self-renewal capacity and multidirectional differentiation potential of SCs offer extensive applications in treating various diseases (Hoang et al., 2022). Numerous studies focus on exploring the effects of SCs on various diseases. Currently, SCs are utilized directly as therapeutic agents, as exosomes, or synergistically with other drugs. For instance, bone marrow mesenchymal stem cells (BMSCs), a type of biomaterial, have shown promising results in cell therapy, demonstrating high safety and low immunogenicity, and can be rapidly applied to treat diseases (Hoang et al., 2022; Lotfy et al., 2023). Stem cell therapy now spans various fields, including cardiovascular diseases (Zhang et al., 2021a), digestive system diseases (Wang et al., 2021), and cancer-related treatments (Barisic and Childs, 2022). However, available data on the safety of autologous or allogeneic mesenchymal stem cells (MSCs) therapy are often preliminary, thus, precise control over SC characterization, production and delivery methods, and therapeutic regimens is still required (Naji et al., 2017).

**FIGURE 1:**
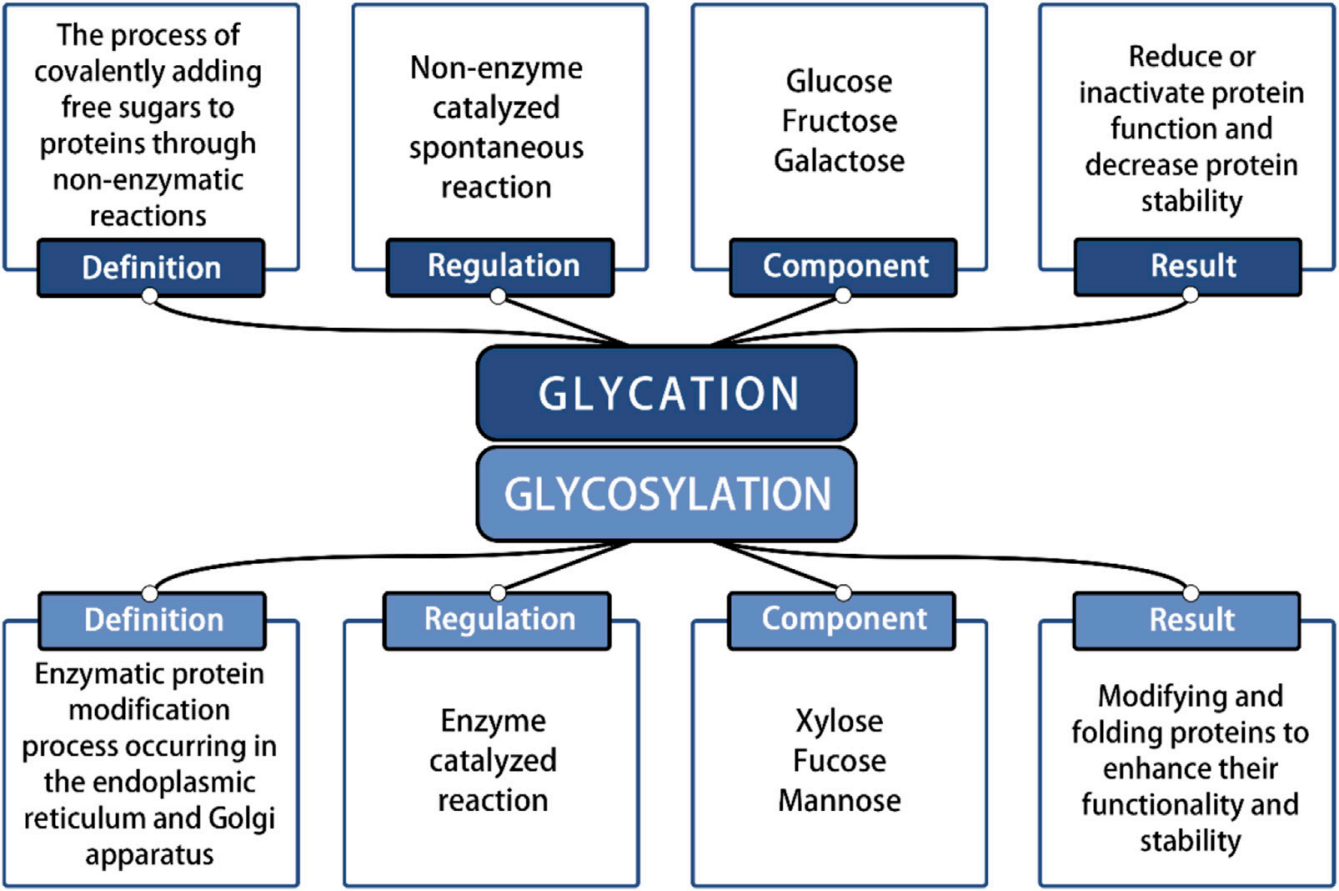
Identification of glycation reaction and glycosylation reaction.

In recent years, significant advancements in stem cell therapy have led to a clearer understanding of its functions and mechanisms, highlighting its immense therapeutic potential. Moreover, various factors influencing the physiological function of SCs have garnered widespread attention and research. Numerous studies have shown that under pathological conditions, the accumulation of Advanced Glycation End Products (AGEs) within the extracellular matrix (ECM) significantly threatens the normal physiological function of SCs (Mouw et al., 2014). This nonenzymatic glycosylation process differs from enzyme-directed glycosylation (Figure 1). It occurs spontaneously between carbohydrates and molecules containing free amino groups, including proteins (Fournet et al., 2018a). AGEs, as nonenzymatic glycation end products, are composed of macromolecules such as proteins, lipids, or nucleic acids and can be classified into two categories: exogenous and endogenous (Singh et al., 2001). AGEs can trigger various pathological mechanisms in the body, including cross-linking with proteins to alter their properties and functions, and activating intracellular signals through receptor and nonreceptor-mediated mechanisms, which increase reactive oxygen species (ROS) and inflammation-related factors (Uribarri et al., 2015). AGEs can accumulate in cells, tissues, and organs throughout the body, leading to oxidative stress and inflammatory responses, and causing detrimental effects on human health. Under the influence of AGEs, the activation of downstream signaling pathways triggers the release of various inflammatory cytokines, which may contribute to the development of diabetes, kidney disease, rheumatoid arthritis, neurodegeneration, cancer and other diseases (Ahmad et al., 2018).

Overall, the cytotoxic effects of AGEs are primarily reflected in irreversible damage to protein structure and functional integrity, resulting from both intermolecular and intramolecular cross-linking. AGEs can cross-link with each other and bind to specific proteins, thereby altering their structure and disrupting their functional properties (Uribarri et al., 2015). This covalent cross-linking leads to the inactivation of biologically active proteins and enzymes, resulting in protein hydrolysis and resistance to digestion. It also creates catalytic sites for ROS formation, thereby exacerbating inflammation and oxidative stress (Wan et al., 2022). Furthermore, AGEs induce various metabolic and biochemical disorders by interfering with intracellular signal transduction processes, and their interactions with different cell surface receptors trigger various cell-mediated pathophysiological responses. For instance, when AGEs bind to the homologous receptor RAGE, they activate multiple downstream signaling pathways, directly affecting the physiological function of SCs (Kume et al., 2005; Uribarri et al., 2015; Ahmad et al., 2018).

This article aims to provide a comprehensive review of how AGEs exert multifaceted effects on the physiological functions of SCs, including their survival, proliferation, differentiation potential, with the goal of exploring the underlying mechanisms in detail. To elucidate the correlation between the physiological function of SCs and the accumulation of AGEs in the ECM, focusing on how AGEs affect the physiological function of SCs. Additionally, we summarize the current methods for addressing the effects of cytotoxic AGEs on SCs. By answering and discussing these questions, we will advance our understanding of the physiological mechanisms and influencing factors of SCs.

## 2 Sources of AGEs

**FIGURE 2:**
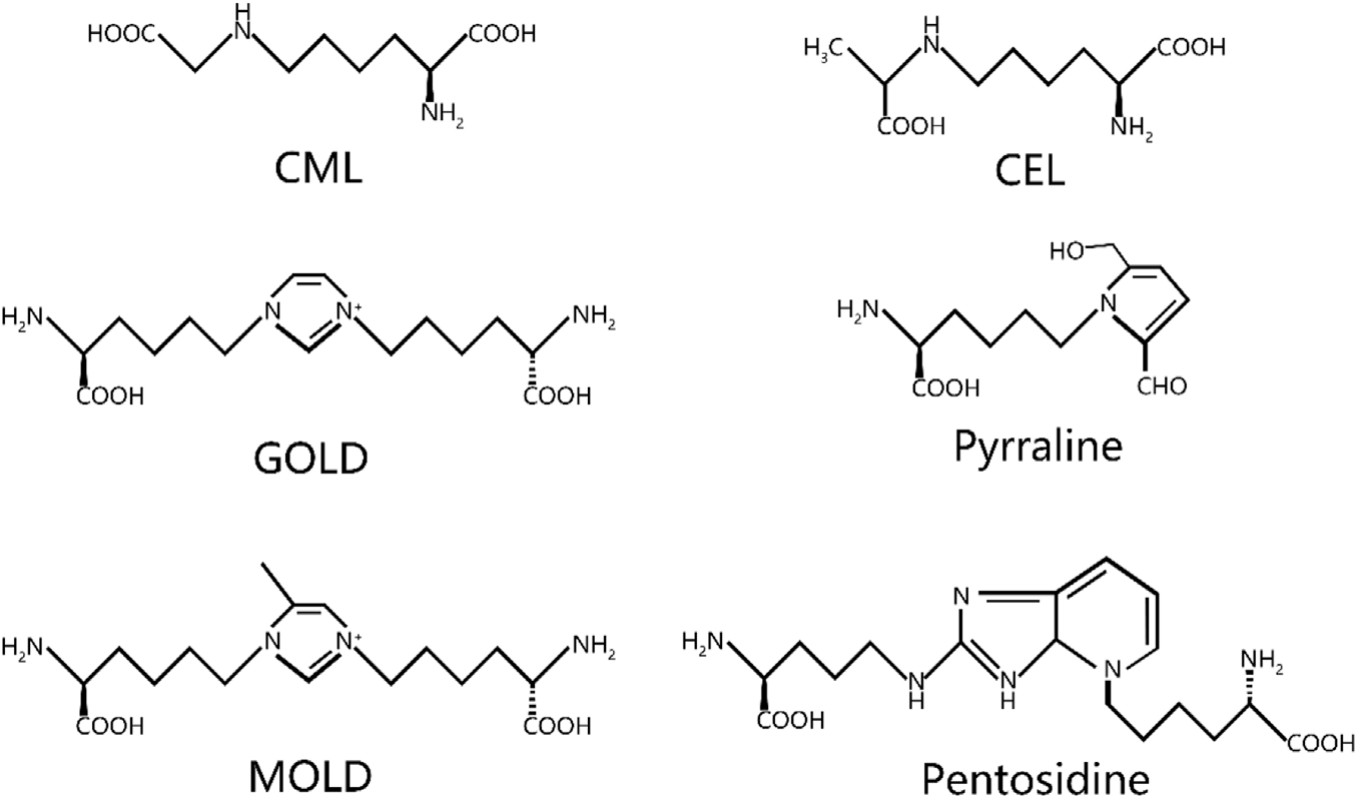
Chemical structure of common AGEs. CML, N-ε-carboxymethyl-lysine; CEL, N-ε-carboxyethyl-lysine; GOLD, glyoxal-lysine dimer; MOLD, methylglyoxal-lysine dimer.

The accumulation of AGEs primarily occurs through two pathways: endogenous and exogenous pathways. Exogenous AGEs are widely present in various foods. The formation of exogenous AGEs is, in fact, closely associated with cooking methods employed in the food industry. Specifically, during food heat treatment, the application of dry heat technologies, such as deep frying, barbecuing, and baking, significantly promotes AGE production. These exogenous AGEs contribute significantly to the total AGEs in the human body (Kellow and Coughlan, 2015). When these AGEs are ingested into the human body through the daily diet, approximately 10%–30% are absorbed and enter the systemic circulation, while the rest are excreted through metabolic pathways (Garay-Sevilla et al., 2021; Khalid et al., 2022). More than 20 AGEs have been identified, with the most common ones being N-ε-carboxymethyl-lysine (CML), N-ε-carboxyethyl-lysine (CEL), pentosidine, pyrraline, glyoxal-lysine dimer (GOLD), methylglyoxal-lysine dimer (MOLD), among others (Figure 2) (Singh et al., 2001).

**FIGURE 3:**
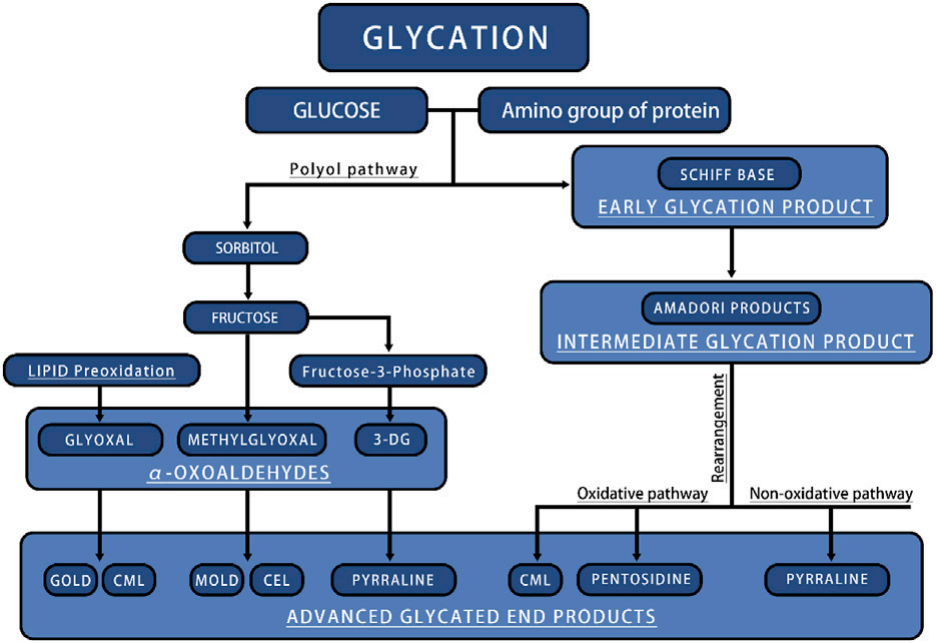
The main process of endogenous AGEs production. CML, N-ε-carboxymethyl-lysine; CEL, N-ε-carboxyethyl-lysine; GOLD, glyoxal-lysine dimer; MOLD, methylglyoxal-lysine dimer.

The formation of endogenous AGEs predominantly occurs via a complex, multistage glycosylation process known as the Maillard reaction. The synthesis of endogenous AGEs involves three steps: 1. The aldehyde group of reducing sugars undergoes nonenzymatic glycation with proteins to form Schiff bases, resulting from the condensation of electrophilic carbonyl groups of reducing sugars with free amino groups; 2. Schiff bases undergo structural rearrangement to produce more stable Amadori products; 3. Amadori products dehydrate and degrade to form AGEs (Xu et al., 2023) (Figure 3). Within an organization, glycation results in protein aggregates forming through three different mechanisms: 1. covalent bonds are formed between AGEs; 2. Oxidation of thiol groups into disulfide bridges; 3. New reactive groups are formed inside proteins. The chemical cross-links created by AGEs contribute to protein network formation and ECM cross-linking, thereby significantly increasing structural rigidity (Fournet et al., 2018b). This nonenzymatic glycosylation process accelerates under hyperglycemic conditions, as commonly seen in diabetes (Stratmann, 2022). The Maillard reaction generates numerous highly reactive carbonyl AGE precursors. Among these precursors, dicarbonyl compounds serve as critical intermediates in carbonyl AGE formation due to their unique chemical properties, playing an indispensable role in the generating endogenous AGEs. In addition, dicarbonyl compounds can be generated through various other reaction pathways and ultimately converted into AGEs. For example, Schiff bases follow the Namiki pathway during oxidation and can be converted into dicarbonyl compounds; Glucose undergoes automatic oxidation through the Wolff pathway under metal catalysis, generating dicarbonyl compounds. Under the oxidation of the acetone pathway, fats also create a series of highly active dicarbonyl compounds. The endogenous production pathways of these dicarbonyl compounds, also known as α-Acetaldehyde, include glucose autooxidation, the polyol pathway, and lipid oxidation. Imbalances in ketone metabolism, especially under hyperglycemic conditions, lead to dicarbonyl stress, a phenomenon particularly common in diabetic patients (Kellow and Coughlan, 2015; Uribarri et al., 2015; Kuzan, 2021) (Figure 3).

## 3 Effects of AGEs on SCs survival and proliferation

AGEs significantly impact the survival and proliferation of SCs, with numerous studies demonstrating their inhibitory effect on SCs proliferation across various sources (Kroemer et al., 2010; Zhang et al., 2021b; Liang et al., 2022; Dobrucki et al., 2024). This effect is closely linked to the impact of AGEs on the ECM. The ECM offers localization and structural support for cells, influencing tissue and organ formation, differentiation, and maintenance by modulating growth factor and receptor levels and regulating the cellular environment’s pH. (Mouw et al., 2014). Blackburn et al. (2017) demonstrated that AGEs significantly impair cell adhesion within the ECM. Specifically, the adhesion ability of BMSCs is significantly diminished when interacting with AGE-modified collagen. This interaction heightens cellular sensitivity to apoptosis, diminishes the progenitor cell population, and impairs SCs differentiation into vascular tissue.

AGEs contribute to apoptosis and senescence in SCs. One of the primary mechanisms of apoptosis involves initiating a cascade of reactions via the activation of cysteine-containing caspases (Cavalcante et al., 2019). In addition to AGEs, RAGE recognizes various ligands, including pro-inflammatory cytokine mediators of the S100/calcogranulin family, high-mobility histone B1 (HMGB1), and the mucopolysaccharide β-amyloid. This nuclear protein is released upon cell necrosis and functions in the extracellular environment. As a pattern recognition receptor, RAGE shares ligands and signaling pathways with many members of the receptor family (Uribarri et al., 2015; Dobrucki et al., 2024). AGE-RAGE binding induces oxidative stress and activates the mitogen-activated protein kinase (MAPK) pathway. MAPK belong to the serine/threonine kinase family, which is not only involved in apoptotic signaling, but also accelerates the process of stem cell apoptosis (Ahmad et al., 2018).

In addition to influencing apoptosis, AGEs also regulate another form of cell death—autophagy. Apoptosis involves the orderly fragmentation of cells into apoptotic bodies, which are swiftly recognized and removed via phagocytosis. Autophagy is an intracellular degradative process in which endogenous or exogenous cytoplasmic components are delivered to lysosomes for degradation. Its primary function is to maintain cell survival and homeostasis by recycling and reusing essential components under stress or nutrient limitation (Kroemer et al., 2010). While autophagy is crucial for cellular homeostasis, excessive autophagy can have harmful effects In Zhang et al. study (Zhang et al., 2021b), it was found that knocking out RAGE can inhibit cell autophagy, indicating that AGEs/RAGE promote autophagy. In Liang et al. (2022) study, it was also found that receptors for AGEs and RAGE are associated with fibrosis and autophagy. Furthermore, inhibiting RAGE provides cardiac protection by reducing hypertrophy and fibrosis in mice. Similarly, Zhang et al. (2023) observed that AGE/RAGE interactions stimulate autophagy.

## 4 Impact of AGEs on the differentiation potential of SCs

The regeneration of tissues is intricately linked to the differentiation of SCs. For instance, during bone development, MSCs migrate to the target site of bone formation, thereby initiating the first stage of bone development. The mechanism of osteogenesis in the human body involves two main pathways: 1. The direct differentiation of cells into osteoblasts, a process known as intramembranous osteogenesis; 2. The indirect pathway, endochondral osteogenesis, involves the differentiation of chondrocytes and their eventual transformation into bone tissue. This osteogenic process ensures normal bone development and formation. Currently, most osteogenic studies focus on osteogenic differentiation as the primary strategy for osteogenic differentiation (Ding et al., 2022). Various mechanisms affect the differentiation process of MSCs, including the AGE/RAGE pathway (Wang et al., 2022), Wnt/β-catenin pathway (Zhang et al., 2018), Notch-Hes1 pathway (Islam and Aboussekhra, 2019), TGFβ pathway (Notsu et al., 2014). The osteogenic differentiation potential of SCs has remarkable plasticity and can be regulated and transformed through a variety of mechanisms. For instance, specific growth factors or pharmacological agents can effectively direct SC differentiation into osteoblasts. These growth factors or drugs direct the transformation of SCs into osteoblasts by interacting with intracellular signaling pathways that regulate gene expression and cellular function (Fu et al., 2021).

### 4.1 AGE/RAGE pathway

Sun et al. (2020) proposed that the AGE/RAGE axis can inhibit the osteogenic differentiation of BMSCs. Their study employed inhibition of the AGE/RAGE axis to mitigate dysfunction in SCs differentiation. Okazaki et al. found that AGEs constrained the osteogenic differentiation of mouse stromal ST2 cells by inhibiting Osterix (OSX) expression and partially increasing RAGE expression. Furthermore, AGEs interfere with the process of SCs differentiation into bone cells by potentially reducing osteocalcin production while increasing RAGE expression (Okazaki et al., 2012). AGEs exert a more pronounced impact during the immature stage of osteoblasts compared to the differentiation stage, inhibiting differentiation and reducing the number of mature osteoblasts (Ogawa et al., 2007). Stolzing et al. (2010) added different doses of AGEs to cultured MSCs and found that the self-renewal and osteogenic differentiation of MSCs were significantly reduced. Under osteogenic differentiation conditions, the extent of this effect depended on the concentration of AGEs in the culture medium. Furthermore, the proliferation of MSCs significantly increased in the low-concentration group, while normal proliferation and osteogenic differentiation of MSCs were impaired in the high-concentration group. They also observed that AGEs suppressed osteocalcin mRNA expression in rat MSCs, thereby hindering their differentiation. Lin et al. (2016) reported that HMGB1 facilitate the osteogenic differentiation of BMSCs while also increasing the expression of RAGE and Toll-like receptors 2 and 4 (TLR2/4) bound to HMGB1. RAGE, a high-affinity receptor for HMGB1, can activate the p38/MAPK and NF-κB pathways upon binding to HMGB1, thereby promoting the osteogenic differentiation of BMSCs (Park et al., 2004). The p38/MAPK pathway plays a crucial role in cell cycle regulation (Barnum and O’Connell, 2014); Kim et al. reported (Kim and Kwon, 2013) that COMP-Ang1 induces the upregulation of the PI3K/AKT and p38/MAPK pathways, thereby facilitating the attenuation of osteogenic differentiation of MSCs by AGEs via the Ang1/Tie2 pathway.

### 4.2 TGF-β pathway

TGF-β is a crucial factor in regulating the differentiation of MSCs and plays a vital role in stem cell differentiation (Li et al., 2024). Notsu et al. considered that AGEs increase TGF-β by binding to RAGE, and the AGE-TGF-β pathway has a negative effect on the differentiation of MSCs into osteoblasts, impairing their differentiation. This indicates that TGF-β is one of the factors influencing the differentiation potential of SCs (Notsu et al., 2014). In recent years, joint cartilage regeneration technology has advanced significantly, driven by continuous improvements in biological scaffold materials. TGF-β3, as an important isoform of the TGF-β family, plays a pivotal role in mesenchymal stem cell differentiation through both Smad-dependent and non-Smad pathways. Its active involvement and tightly regulated role in the bone healing process have been widely recognized. In recent years, there has been increasing interest in the potential of TGF-β3 to promote and induce the proliferation, osteogenesis, and chondrogenic differentiation of adult SCs in biological scaffold materials. In particular, the induction of TGF-β3 is particularly significant in the early stages of the osteogenic process, providing new therapeutic strategies and research ideas for bone tissue regeneration. These studies not only help us to understand the mechanism of cartilage repair and regeneration deeply, but also provide a solid theoretical basis and experimental foundation for future clinical applications (Li et al., 2018; Roth et al., 2019; Martin et al., 2021).

In general, the role of TGF-β3 in cartilage formation is cell-type specific. Jin et al.'s reported that the inhibitory effect of TGF-β3 on chondrocytes is achieved through the activation of Notch signaling, which inhibits the proliferation of mesenchymal cells and pre-cartilage condensation (Jin et al., 2007). In another study, they also reported a similar finding regarding the inhibitory effect of TGF-β3 on the differentiation of MSCs, which is that TGF-β3 downregulates Protein Kinase C-α (PKC-α) mediated activation of connexin 43, integrin β4, and ERK, inhibiting chondrogenic differentiation of mesenchymal cells (Jin et al., 2008). In contrast to the inhibitory effect of TGF-β3 on MSC differentiation mentioned above, Zheng et al. found that knocking out the TβRIII gene can promote TGF-β3-induced MSCs cartilage differentiation, demonstrating the positive induction effect of TGF-β3 on mesenchymal stem cell differentiation (Zheng et al., 2018). Similarly, Jin et al. found that TGF-β3 stimulates the differentiation of MSCs into chondrocytes and inhibits the differentiation of chondrocytes. This is because TGF-β3 promotes chondrogenic differentiation of mesenchymal cells by activating the PKC-α and p38 MAPK pathways (Jin et al., 2006). Based on the multiple studies on TGF-β3 specified above, it can be concluded that the differences in TGF-β3 are due to its various functions, manifested as a mixed effect of induction and inhibition on the differentiation process of MSCs.

Overall, it is crucial to investigate the complex signaling pathways and mechanisms by which AGEs affect the differentiation process of SCs. This will not only aid in revealing the mechanisms by which AGEs influence SC differentiation, but also provide insight into potential strategies to reverse the toxic effects of AGEs.

### 4.3 Wnt/β-catenin pathway

The Wnt/β-catenin pathway, a central signaling pathway, precisely regulates cell polarity, determines the differentiation fate of cells, guides the migration process of cells, and has a profound impact on spindle formation, organ development, and stem cell renewal (Nayak et al., 2016). Currently, 19 Wnt ligands have been identified, and all of these ligands specifically bind to a seven-transmembrane Wnt receptor named Frizzled (FZD) (Houschyar et al., 2019). The Wnt pathway, a well-established osteogenic differentiation pathway, is a complex system comprising three distinct pathways, which are believed to be activated upon Wnt receptor activation: the canonical Wnt/β-catenin cascade, the noncanonical planar cell polarity (PCP) pathway, and the Wnt/Ca2+ pathway.

**FIGURE 4:**
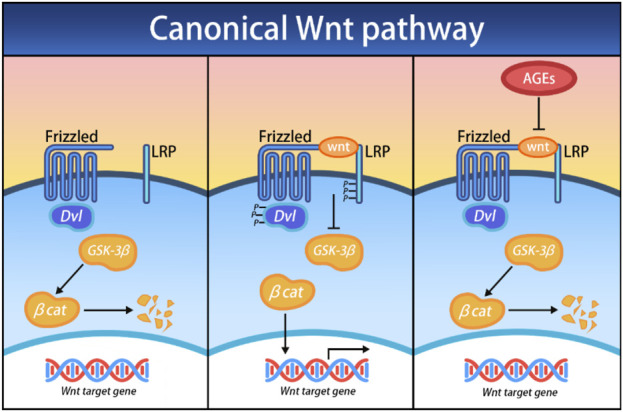
Effects of AGEs on the canonical Wnt/β-catenin cascade; Dvl, Dishevelled; GSK-3β, glycogen synthase kinase 3 beta; βcat, β-catenin.

In the canonical Wnt/β-catenin cascade, the central event is the nuclear translocation of the β-catenin protein and its regulation of target genes. In the absence of Wnt ligands, β-catenin is degraded by intracellular complexes-primarily composed of glycogen synthase kinase 3 (GSK-3). However, once the canonical Wnt/β-catenin cascade is activated, Dishevelled proteins (Dvl) are triggered, which in turn inhibit GSK-3, thereby stabilizing β-catenin and promoting its nuclear translocation and target gene expression. Atypical Wnt signaling also plays a crucial role in the differentiation of bone tissue. Unlike the canonical Wnt/β-catenin cascade, the atypical Ca2^+^ dependent Wnt pathway uniquely promotes osteogenesis in MSCs. When the Wnt ligand binds to the FZD receptor, it activates G proteins, triggering the release of Ca2^+^ ions from the endoplasmic reticulum. This process initiates the Protein Kinase C (PKC) pathway and continue signaling to promote osteogenesis (Ahmadi et al., 2022). The Wnt pathway is initiated when Wnt ligands bind to FZD receptors, activating G proteins that subsequently trigger the release of Ca2^+^ ions from the endoplasmic reticulum, which then initiates the PKC pathway. Low-density lipoprotein receptor associated protein 5/6 (LRP5/6) or receptor tyrosine kinase-like orphan receptors (RORs) function as common receptors alongside FZD, facilitating the binding of Wnt proteins to their receptors. The involvement of these co-receptors dictates the downstream effects following successful ligand binding, initiating either the canonical Wnt/β-catenin cascade or the noncanonical planar cell polarity (PCP) pathway (Houschyar et al., 2019; Ahmadi et al., 2022) (Figure 4). Zhou et al. (2023) found that AGEs can impair the osteogenic differentiation process of BMSCs by upregulating the expression of fat and obesity-related gene FTO. This process is regulated by FTO to modify the SOST transcript with m6A, increase the mRNA stability recognized by YTHDF2, inhibit the Wnt signaling pathway, and ultimately disrupt the differentiation of BMSCs into bone. Minear et al. (2010) amplified the cell Wnt response by removing the Axin 2 gene in a mouse model and found that delivering liposome vesicles containing purified Wnt-3a protein can promote the Wnt pathway, leading to increased proliferation and early differentiation of BMSCs, thereby accelerating fracture healing.

Growth factors activate aspects of the Wnt pathway. The TGF-β pathway is a membrane-to-nucleus signaling cascade activated by receptor-mediated transcription factors. Due to structural and functional considerations, the 32 family members are classified into TGF-β and bone morphogenetic proteins (BMPs) subfamilies, along with other variations (David and Massagué, 2018). BMPs have been extensively studied, with BMPs 2, 6, and 9 being the primary isoforms. As potent growth factor, BMPs stimulate MSCs to differentiate into osteoblasts (Carreira et al., 2014). The functional Wnt signaling pathway constitutes the core mechanism of BMP-induced osteogenic differentiation of MSCs. There is a significant interaction between the Wnt and the TGF-β pathways, as they share some key regulatory targets, thus forming a complex signaling network. Among them, β-catenin, a key node in this network, plays a crucial role in regulation (Case and Rubin, 2010). β-catenin plays various roles during different phases of bone repair. In the initial stages following injury, it modulates the osteoblast-to-chondrocyte ratio within the callus tissue induced by MSCs, ensuring a balanced and coordinated repair process (Bao et al., 2017). In the later stages of bone healing, β-catenin induces osteoblasts to differentiate and produce an osteogenic matrix, promoting bone reconstruction and regeneration (Wang et al., 2017). Zhang et al. (2009) suggest that BMP2 regulates β-catenin by stimulating the expression of Lrp5 in osteoblasts and inhibiting the expression of β-Trcp. Chen et al. (2019) also found that the key growth factor BMP2 stimulates the Wnt/β-catenin pathway to promote the osteogenic differentiation of BMSCs. The addition of Wnt-3a enhances the osteogenic effect of BMP9. However, it is counteracted by the downregulation of β-catenin or the increased expression of FrzB, which acts as an antagonist of the FZD receptor (Boland et al., 2004).

## 5 Strategies for dealing with the toxic effects of AGEs on SCs

Effective intervention in AGEs-induced damage is critical for promoting the normal physiological activity of SCs. Given the central role of AGEs in stimulating tissue fibrosis and mediating matrix cross-linking, strategies such as reducing AGEs formation, enhancing AGEs degradation, and blocking AGEs cross-linking show promise as therapeutic approaches. Currently, interventions targeting AGEs focus on blocking the pathways through which they exert their effects, thereby mitigating the deleterious impact of AGEs on SCs’ physiological functions. In this process, AGE/RAGE and Wnt/β-catenin signaling pathways have become the focus of our attention, and they provide important clues for us to understand the mechanism of AGEs and develop effective interventions.

### 5.1 Targeting the AGE/RAGE pathway

The glyoxalase system is integral, serving as a key enzyme system present in all mammalian cells. This system consists of two enzymes that act in concern: glyoxalase 1 (Glo1) and glyoxalase 2 (Glo2). The AGE/RAGE pathway alleviate differentiation dysfunction of BMSCs by enhancing the activity of Glo1. AGEs activate complex signaling pathways by binding to RAGE, thereby triggering various toxic effects in the organism. These enzymes catalyze sequential reactions, with reduced glutathione (GSH) serving as a catalytically active and essential component. The Glo1 enzyme plays a crucial role in the metabolic process by catalyzing the nonenzymatic isomerization of the active dicarbonyl metabolite methylglyoxal (MG) with glutathione to produce dithiol acetaldehyde. Additionally, Glo2 catalyzes the hydrolysis of S-D-lactoylglutathione to generate D-lactic acid, thereby efficiently supplementing the glutathione consumed in the Glo1-catalyzed process. Due to this synergistic action, the glyoxalase system is able to efficiently process dicarbonyl compounds *in vivo* and maintain normal metabolic functions of cells (Rabbani and Thornalley, 2019). Wang et al. (2019) used glycine to inhibit the formation of AGEs, and the study described that Glo1 also mediates this effect. In Jandial et al. mouse model (Jandial et al., 2018), blocking Glo1 resulted in increased AGE production and upregulation of RAGE expression. Consequently, Glo1 inhibition caused cellular accumulation of MG, triggering rapid modifications of proteins, lipids, and DNA, ultimately inducing apoptosis. To counteract the adverse effects of AGEs on primary SCs, an effective strategy involves blocking the interaction between AGE and RAGE. The discovery of Zhang et al. can be utilized with the RAGE inhibitor FPS-ZM1, which can attenuate the adverse effects of AGEs on the osteogenic potential of SCs (Zhang et al., 2018). Rasheed et al. (2011) found that knocking down RAGE by pre-treating soluble RAGE (sRAGE) or using siRNAs effectively reduced the cytotoxicity of AGEs. Based on the above research, activating Glo1 or directly inhibiting the binding of AGEs to RAGE is a highly feasible strategy to mitigate the toxic effects of AGEs.

### 5.2 Promoting DNA demethylation

Alterations in the Wnt/β-catenin pathway significantly influence bone metabolism. Notably, elevated concentrations of Wnt3a can inhibit the osteogenic differentiation of BMSCs, indicating that regulating the Wnt/β-catenin pathway needs to be fully balanced to avoid adverse effects on stem cell differentiation. Therefore, comprehensive research on the Wnt/β-catenin pathway and its interaction with DNA methylation processes is highly important for optimizing the physiological functions of SCs in AGEs -induced environments (Boland et al., 2004). In Liang et al.'s study, DNA methylation was found to have a substantial impact on the expression of Wnt/β-catenin signaling pathway genes, which also proves that the impact of DNA methylation on the physiological function of MSCs is achieved through the Wnt/β-catenin pathway (Liang et al., 2015). DNA methylation is a molecular modification that determines cell identity and lineages by regulating gene expression and maintaining genomic stability. Under the action of DNA methyltransferase, the covalent bond at the cytosine 5 carbon position of the CpG dinucleotide in the genome binds to a methyl group. DNA methylation induces changes in chromatin structure, DNA conformation, stability, and the dynamics of DNA-protein interactions, thus exerting control over gene expression (Nishiyama and Nakanishi, 2021). DNMT1, DNMT3a and DNMT3b play indispensable roles in DNA methylation. Recent research by Zhang et al. (2018) demonstrated that the expression of DNMT1 and DNMT3a was upregulated, indicating that AGEs increased the level of DNA methylation in ADSCs. To reverse this effect, the investigators used FPS-ZM1, which successfully rescued the loss of osteogenic differentiation in ADSCs by inhibiting AGEs induced DNA methylation. In a study by Li et al. (2020), when ADSCs were cultured in a medium containing AGEs, they exhibited high levels of 5-mC and DNMTs, accompanied by a significant reduction in osteogenic differentiation capacity *in vitro*. However, by applying DNMT inhibitors (5-aza-dC), investigators found that the osteogenic differentiation potential of ADSCs was improved. The promotion of DNA demethylation enhanced the osteogenic differentiation of ADSCs, highlighting the critical role of DNA methylation levels in regulating this process.

## 6 Conclusion

Both endogenous and exogenous AGEs negatively affect the physiological function of SCs. These strong oxidants continuously weaken the cell’s natural defense mechanisms, leading to abnormal oxidative stress and inflammatory responses. However, this unfavorable situation is not irreversible and stem cell therapy is a potential coping strategy to curb the damage caused by AGEs effectively. Elucidating the underlying mechanisms of the impact of AGEs on stem cell toxicity and devising pertinent solutions are vital for advancing stem cell therapy technology.

## Abbreviations

AGEs, Advanced Glycation End Products; SCs, stem cells; MSCs, mesenchymal stem cells; hPSCs, human pluripotent stem cells; BMSCs, bone marrow mesenchymal stem cells; ECM, extracellular matrix; ERK, extracellular signal-regulated kinases; CML, N-ε-carboxymethyl-lysine; GOLD, glyoxal-lysine dimer; MOLD, methylglyoxal-lysine dimer; HMGB1, high-mobility histone B1; FZD, Frizzled; GSK-3, glycogen synthase kinase 3; Dvl, Dishevelled proteins; NF-κB, Nuclear Factor Kappa B; COMP-Ang1, cartilage oligomeric matrix protein-angiopoietin-1; PKC, Protein Kinase C; PCP, planar cell polarity; p38/MAPK, p38 mitogen-activated protein kinase; LRP5/6, Low-density lipoprotein receptor associated protein 5/6; RORs, receptor tyrosine kinase-like orphan receptors; ROS, reactive oxygen species; OSX, osterix; TLR2/4, Toll-like receptors 2 and 4; BMPs, bone morphogenetic proteins; PI3K/AKT, phosphatidylinositol 3-kinase/protein kinase B; RAGE, Receptor for advanced glycation end products; sRAGE, soluble RAGE.
